# A genetic predisposition score for muscular endophenotypes predicts the increase in aerobic power after training: the CAREGENE study

**DOI:** 10.1186/1471-2156-12-84

**Published:** 2011-10-03

**Authors:** Tom Thomaes, Martine Thomis, Steven Onkelinx, Robert Fagard, Gert Matthijs, Roselien Buys, Dirk Schepers, Véronique Cornelissen, Luc Vanhees

**Affiliations:** 1 Cardiovascular Rehabilitation Unit, Department of Rehabilitation Sciences, Katholieke Universiteit Leuven, Tervuursevest 101, 3001 Heverlee, Belgium; 2 Research Centre for Exercise and Health, Department of Biomedical Kinesiology, Katholieke Universiteit Leuven, Tervuursevest 101, 3001 Heverlee, Belgium; 3 Hypertension and Cardiovascular Rehabilitation Unit, Department of Cardiovascular Diseases, UZ Leuven, Herestraat 49, 3000 Leuven, Belgium; 4 Department of Human Genetics, Centre for Human Genetics of the University Hospitals, UZ Leuven, Herestraat 49, 3000 Leuven, Belgium

**Keywords:** Cardiac Rehabilitation, Ischemic Heart Disease, Exercise Test, Physical capacity, genetic association, aerobic power

## Abstract

**Background:**

It is widely accepted that genetic variability might explain a large part of the observed heterogeneity in aerobic capacity and its response to training. Significant associations between polymorphisms of different genes with muscular strength, anaerobic phenotypes and body composition have been reported. Muscular endophenotypes are positively correlated with aerobic capacity, therefore, we tested the association of polymorphisms in twelve muscular related genes on aerobic capacity and its response to endurance training.

**Methods:**

935 Coronary artery disease patients (CAD) who performed an incremental exercise test until exhaustion at baseline and after three months of training were included. Polymorphisms of the genes were detected using the invader assay. Genotype-phenotype association analyses were performed using ANCOVA. Different models for a genetic predisposition score (GPS) were constructed based on literature and own data and were related to baseline and response VO_2 _scores.

**Results:**

Carriers of the minor allele in the R23K polymorphism of the glucocorticoid receptor gene (*GR*) and the ciliary neurotrophic factor gene (*CNTF*) had a significantly higher increase in peakVO_2 _after training (p < 0.05). Carriers of the minor allele (C34T) in the adenosine monophosphate deaminase (*AMPD1*) gene had a significantly lower relative increase (p < 0.05) in peakVO_2_. GPS of data driven models were significantly associated with the increase in peakVO_2 _after training.

**Conclusions:**

In CAD patients, suggestive associations were found in the *GR, CNTF *and the *AMPD1 *gene with an improved change in aerobic capacity after three months of training. Additionally data driven models with a genetic predisposition score (GPS) showed a significant predictive value for the increase in peakVO_2_.

## Background

In patients with ischemic heart disease aerobic power and skeletal muscle strength are often reduced in comparison with age and sex-matched healthy subjects [1]. Regular physical activity improves aerobic power and skeletal muscle strength and is accompanied with an increase in survival in these patients. Moreover, aerobic power has been shown to be an independent prognostic factor of survival in patients with heart disease [2-4]. Therefore, participation in a cardiovascular exercise training program is strongly recommended in coronary artery disease (CAD) patients [5-7]. Recently, we reported an average increase in aerobic power of 26% ± 22% (Standard Deviation; SD) after a training period of three months in 1909 CAD patients [8]. A considerable interindividual variation could be observed with regard to aerobic power and its response to physical training. We identified 12 independent determinants, which accounted for 21% of the total variation in the aerobic power response to three months of training [8]. However, a large part of the individual variation remained unexplained. Evidence from healthy families and twin studies showed that a large part (50 to 66%) of the interindividual variation in aerobic power and its training response can be explained by genetic factors [9-11]. Likewise, multivariate genetic analysis on muscle strength phenotypes indicated that additive genetic factors could explain 66-78% of the variance in maximal elbow flexor torque [12]. Dynamic resistance training programs induce changes in muscle fibre size, fibre type and muscle metabolism [13-16] which can lead to a greater oxygen uptake by the muscle and a higher peak oxygen uptake (peakVO_2_) [17]. Conley et al. [18] already pointed out that muscle volume is an important determinant in the response of peakVO_2 _to training. Therefore by increasing the amount of muscle mass used in the various exercises, independently of the mode of (resistance) training, the haemodynamic responses become more dynamic (aerobic) in nature. This was confirmed by Longhurst et al. [19] who attributed the slight increase of peakVO_2 _in weight lifters to the dynamic components in the exercises. In a subset of 80 CAD-patients we observed a significant increase (10-15%) in muscle force after three months of cardiac rehabilitation [20]. In agreement with Kostka et al. [21] we reported a correlation of r = 0.60 between baseline peakVO_2 _and isometric and isokinetic muscle strength. Further, a correlation of r = 0.32 between change in peakVO_2 _and change in isometric muscle force could be observed [20]. We therefore hypothesized that genes encoding for the different muscular subsystems, e.g. muscular structure, metabolism, cytokines, growth or differentiation factors, neurotrophic factors and hormones might be biologically plausible candidate genes that could be involved in aerobic power variability and its response to training. However, so far, research has mainly focussed on the associations between polymorphisms of these various genes and muscle strength, muscle function or their response to training and not on the association with aerobic power [22,23].

We therefore aimed to identify whether previously described polymorphisms in these genes account for differences in aerobic power or its response to training in a cohort of 935 cardiac patients included in the CAREGENE study (CArdiac REhabilitation and GENetics of Exercise performance and training effect). The significance of a genetic predisposition score (GPS) for 7 literature based replicated strength-related polymorphisms was tested in predicting baseline aerobic power. Additionally, data driven models for GPS were tested in predicting baseline aerobic power and the response of peakVO_2 _to training.

## Methods

### Study population

A detailed description of the CAREGENE study design, eligibility criteria and methodology are presented elsewhere [24]. In brief, 935 biologically unrelated Caucasian CAD patients (76 women; mean age 56 ± 0.3 yrs) who had performed a graded cycle ergometer test until exhaustion, before and after three months of physical training, were included. The study protocol was approved by the Ethical Committee of the Faculty of Medicine of the Catholic University of Leuven and written informed consent was obtained from each participant.

### Exercise training

Patients completed a three months ambulatory supervised exercise training program (90 minutes/session and involving cycling, running, arm ergometry, rowing, predominantly isotonic calisthenics and relaxation). Training frequency averaged 2.27 ± 0.02 times/week and training intensity was 79.7 ± 0.35% of the maximal heart rate (HRmax). The latter was calculated as: (training heart rate/peak heart rate) * 100, where the mean training heart rate of the last 3 exercise sessions and peak heart rate of the exercise test after training were used.

### Exercise testing

A detailed description of the exercise test methodology has been reported previously [24]. Before and after the three months training program patients completed a maximal graded exercise test on a cycle ergometer with respiratory gas analysis. The initial workload of 20 W was increased until exhaustion by 30 W every 3 min and after a protocol change in the year 2000 the initial workload of 20 W was increased by 20 W per min. Heart rate was measured throughout the test with a 12-lead ECG and blood pressure was measured every 2 minutes. Respiratory data were measured through breath-by-breath analysis. PeakVO_2 _was defined as the highest 15 seconds average of VO_2 _obtained at the end of the test and was expressed as mL.min^-1^. The percent of predicted peakVO_2 _was calculated as peakVO_2 _divided by maximal predicted VO_2_, using the values reported by Wasserman et al. [25].

### Genotype determinations

21 single nucleotide polymorphisms (SNPs) in 12 muscular related genes were genotyped (Table 1). Selection of genes and SNPs was based on a literature search at the beginning of the study. SNPs had to be associated with muscular phenotypes or aerobic phenotypes. DNA was extracted from white blood cells using the 'salting-out' method and the Invader TM assay (Third Wave Technologies) was used for genotyping [26]. The Invader assay combines structure-specific cleavage enzymes and a universal fluorescent resonance energy transfer (FRET) system. When the gene-specific probes bind the target, these enzymes will cleave. This mechanism warrants the specificity for distinguishing between alleles, whereas the FRET system generates an amplified readout [27]. In every experiment synthetic target oligonucleotides were used as controls. Third Wave Technologies designed and provided all reaction components. Genotyping was performed in a 96-well format. By combining Probe mix (211 μl), Cleavase enzyme (79 μl) FRET mix (317 μl) and MAP buffer (26 μl) the reaction mixture was prepared. Of this mixture 6 mL was added into a 96-well plate. Six micro litres of no target blank, synthetic target oligonucleotides, or genomic DNA samples (50 ng/μl) were added. After short centrifugation and incubation at 63°C for four hours, fluorescent intensities were measured using a fluorescence microtiter plate reader. By calculating the ratios of the net wild type and net mutant (or minor allele) signals [28], genotypes were determined. The analysis was repeated once on those samples where no genotype could be obtained during initial testing. Samples were excluded from the analysis when genotyping failed twice (this failure rate varies between 1.4% and 5.1%, data not shown).

**Table 1:** Overview of the investigated genes and polymorphisms within muscular structural or functional subsystems

Sub-System	Gene	Polymorphism	Genotype frequency (n patients)		
Metabolism	adenosine monophosphate deaminase (*AMPD1*)	C34T/rs17602729	CC (652)	CT (239)	TT (24)

Muscular structure	Alpha-actinin 3 (*ACTN3*)	Q523R/rs1671064	TT (303)	TC (444)	CC (176)
		R577X/rs1815739	GG (468)	GA (386)	AA (66)
	
	Myosine light chain kinase	C49T/rs2700352	GG (589)	AG (289)	AA (38)
	(*MLCK*)	C37885A	GG (749)	GT (157)	TT (9)

Cytokines	Interleukin 6 (*IL-6*)	G-174C/rs1800795	GG (332)	GC (411)	CC (176)
	
	Interleukin 15 receptor	PstI/rs2296135	CC (238)	CA (468)	AA (206)
	alpha (*IL-15Ra*)	BstNI/rs2228059	TT (223)	GT (480)	GG (211)
		HpaII/rs3136618	GG (227)	GA (459)	AA (203)

Growth or differentiation factors	Insulin-like growth factor 2 (*IGF-II*)	ApaI/rs680	GG (448)	AG (388)	AA (80)
	
	Activin-type II receptor B (*ACVR2B*)	Rs2268757	TT (283)	CT (468)	CC (162)
	
	Follistatin (*FST*)	Rs3756498	CC (605)	CT (278)	TT (30)
		Rs12152850	CC (606)	CT (278)	TT (35)
		Rs12153205	TT (601)	CT (275)	CC (36)
	
	v-akt murine thymoma viral oncogene homolog 1 (*AKT1*)	G205T/rs1130214	GG (452)	GT (377)	TT (76)

Neurotrophic factors	Ciliary Neurotrophic Factor (*CNTF*)	G-6A/rs1800169	GG (664)	GA (226)	AA (21)
	
	Ciliary Neurotrophic	C-1703T/rs3808871	CC (580)	CT (300)	TT (41)
	Factor Receptor (*CNTFR*)	C174T	CC (695)	CT (187)	TT (12)

Hormones	Glucocorticoid Receptor	R23K/rs6190	GG (857)	GA (54)	AA (1)
		N363S/rs6195	AA (843)	AG (70)	GG (2)
		BclI/rs41423247	CC (399)	GC (403)	GG (120)

Genotype frequencies (N) in the CAREGENE study included in brackets

### Statistical methods

Data were analyzed using SAS statistical software ^® ^version 9.1 for Windows (SAS Institute Inc, Cary, NC, USA). Data are reported as means ± SE or as number of patients with percentage for dichotomous variables. To test whether the observed genotype frequencies were in Hardy-Weinberg equilibrium a χ^2^- test with one degree of freedom was used. Distributions were checked for normality with the Shapiro-Wilk statistic. Comparisons between the exercise test at baseline and after training were made by paired Student's t-test; comparisons across genotypes by AN(C)OVA, followed by Fisher's protected least significance difference (LSD) method if significant. Where appropriate, categorical data were tested by χ^2 ^or by Fisher's exact test. ANCOVA was used with age, gender, stature, body mass and baseline peakVO_2 _as covariates to test potential relationships between gene variation and the increase in peakVO_2 _after training. Gene polymorphisms with a minor allele frequency of less than 2% were analysed as carriers vs. non-carriers of the minor allele.

Given the large set of gene variants and 3 phenotypes under study, multiple testing issues probably induce false positive interpretation of association findings. To partially overcome the multiple testing problem and to test for the additive effect of cumulative (response in) VO_2 _uptake- alleles, a genetic predisposition score (GPS) was calculated and its predictive value tested. Based on recent literature, seven polymorphisms were selected for this 'increasing allele' approach for baseline peakVO_2 _[29-31]. Each of these polymorphisms has been associated with muscle strength or function in at least three independent replicated studies with evidence for the same increasing allele in all studies. The selected polymorphisms were Alpha-actinin 3 (*ACTN3 *R577X), Myosine light chain kinase (*MLCK *C49T), Insulin-like growth factor 2 (*IGF-II *ApaI), Glucocorticoid receptor (*GR*, R23K), Interleukin 15 receptor alpha (*IL15Ra *BstnI), Ciliary Neurotrophic Factor Receptor *(CNTFR *C-1703T and C174T). Additional file 1 shows which alleles were chosen as the 'increasing alleles'. An additive effect was hypothesized and equal weights were given for each increasing allele, because no well-defined effect sizes are known for the different SNPs and weighting of increasing alleles might only have limited effect [32]. The genetic predisposition scores was calculated for each individual by adding the strength-increasing alleles (0, 1 or 2) from all seven variants and was analyzed in a GLM approach with age, gender, stature and body mass as covariates. Additionally, based on our own data, backward regression analysis was applied to detect three subsets of SNPs to be associated with the three phenotypes; baseline peakVO_2_, the simple difference (ml/min) in peakVO_2 _(ml/min) and the relative difference (%) in peakVO_2 _after training. Based on these subsets of variants we created three models to test the predictive value of its GPS. Finally, when the predictive value of the GPS was significant in the model, patients were categorized in the upper 10% peakVO_2 _(High group) and lower 10% peakVO_2 _(Low group) using sex-specific cut-off scores. Similarly, high and low responder groups were defined. Different logistic regression models were used to test the power of the GPS to predict 'high group' status, and to produce the receiver operating characteristic curve (ROC curve) and the area under the receiver operating characteristic curve (AUC). Predicted probability was calculated for a hypothetical average patient. Adjusted odds ratios were calculated to estimate the effect of the GPS score as the odds per increasing allele to belong to the high peakVO_2 _group or to the high responder group. All statistical tests were performed two-sided at a significance level of 5%.

## Results

Descriptive characteristics of the overall study cohort (n = 935) are presented in Table 2. At baseline, overall aerobic power averaged 1716 ± 16 mL.min^-1 ^or 77.9 ± 0.6% (± SE) of the predicted normal value (men: 1766 ± 16 ml/min, women: 1142 ± 31 ml/min, p < 0.0001). Three months of physical training resulted in a significant average increase of the aerobic power by 24.2 ± 0.6% (p < 0.001) (men: 24.1 ± 17.4%, women: 25.1 ± 21.1, p = 0.47), ranging from a decrease of 33.6% to an increase of 111.1%. Baseline peakVO_2 _and its change after training follow the normal Gaussian distribution (p > 0.05). At peak exercise, respiratory gas exchange ratio averaged 1.14 ± 0.003 at baseline and 1.13 ± 0.002 after training (p > 0.05). Peak ventilatory equivalent for oxygen was 38 ± 0.2 and 37 ± 0.2 respectively (p > 0.05). None of the patient characteristics and training characteristics were significantly different amongst the different polymorphisms. χ^2 ^tests revealed no deviations from the Hardy-Weinberg equilibrium except for G-174C of the *IL-6 *gene (p = 0.017).

**Table 2:** Clinical characteristics for biologically unrelated Caucasian CAD patients (n = 935) in the CAREGENE study

Variable	Overall Cohort
	N (%)
Women	76 (8)
Age (years)	56 ± 0.3
Body mass index (kg.m^-2^)	25.8 ± 0.1
History of diabetes	49 (5)
History of hypertension	251 (27)
Current smoking	45 (5)
Past smoking	681 (73)
Complaints of angina pectoris in daily life	41 (4)
dyspnoea in daily life	149 (16)
AMI	630 (67)
-Anterior	252 (27)
-Inferior	333 (36)
CBS	377 (40)
PCI	470 (50)
Angina pectoris	23 (2)

AMI, acute myocardial infarction; CBS, coronary bypass surgery; PCI, percutaneous coronary Intervention. Some patients had more than one pathology. Data are presented as means ± SE for continuous variables and as numbers (percentage) for dichotomous variables.

### Single polymorphism associations

Significant associations with the simple difference (ml/min) and/or relative difference (%) in aerobic capacity after three months of training could be observed for 3 out of the 21 investigated polymorphisms which were genotyped (Table 3 and Additional file 2, Table S1).

**Table 3:** Genotype-phenotype association analysis for muscular subsystem gene polymorphisms and baseline aerobic power and changes after training in the CAREGENE study

Gene	Polymorphism	Allele	Frequency	VO_2_pre	p-value	**VO**_**2 **_**change**	p-value	**VO**_**2 **_**change**	p-value
			N (%)	**(ml/min) **^**a**^		**(ml/min) **^**b**^		(%) b	
**AMPD1**	C34T/rs17602729	CC	652 (71)	1711 ± 15	p = 0.40	396 ± 10	p = 0.11	24.8 ± 0.6	p = 0.04
		CT + TT	263 (29)	1734 ± 23		367 ± 16		22.4 ± 0.99	
									
**CNTF**	G-6A/rs1800169	GG	664 (73)	1724 ± 15	p = 0.43	369 ± 9	p = 0.001	23.1 ± 0.6	p = 0.002
		GA	226 (25)	1701 ± 25		416 ± 16		25.1 ± 1.0	
		AA	21 (2)	1801 ± 81		519 ± 52		33.9 ± 3.3	
									
**GR**	R23K/rs6190	GG	857 (94)	1715 ± 13	p = 0.08	383 ± 9	p = 0.02	23.8 ± 0.6	p = 0.04
		GA + AA	55 (6)	1805 ± 50		464 ± 34*		28.5 ± 2.2*	

AMPD1, Adenosine monophosphate deaminase; CNTF, Ciliary neurotrophic factor; GR, Glucocorticoid receptor

a. Mean ± SE, corrected for gender, age, height and weight;

b. Mean ± SE, corrected for gender, age, height, weight and baseline peakVO_2_

In the R23K polymorphisms of the *GR *gene, carriers of the minor allele had a significantly larger increase in peakVO_2_, both in simple difference (ml/min) (p < 0.05) and in relative (p < 0.05) term, compared to the homozygous genotype groups. Further, homozygous CC genotype group members for the C34T polymorphism of the *AMPD1 *gene showed a significantly larger relative increase in peakVO_2 _compared to the carriers of the minor allele (p < 0.05), whereas the increase in peakVO_2 _was significantly larger in the AA genotype group of the G-6A polymorphism of the *CNTF *gene compared to the homozygous GG group (p < 0.01).

### Genetic predisposition score

**Figure 1:**
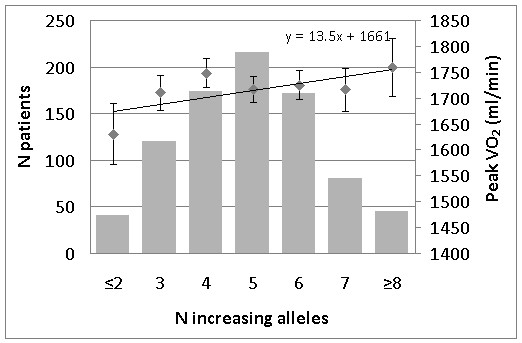
**Genetic predisposition score for muscular subsystems gene polymorphisms (literature based) and baseline peakVO_2 _in the CAREGENE study**. Left Y-axis: Number of patients in each increasing alleles group (bar graph). Right Y-axis: Baseline peakVO_2 _for each increasing alleles group (square dots ± SE) corrected for age, gender, height and body mass. X-axis: GPS - Number of increasing alleles. Regression line for baseline peakVO_2_

A theoretical maximal GPS of 14 increasing alleles could occur for seven selected polymorphisms based on recent literature. 848 patients had complete data for all of these polymorphisms. Figure 1 shows the distribution of GPS within our patient group with four patients exhibiting only one increasing allele up to six patients having nine increasing alleles (max GPS = 9). To avoid the possibility of false positive results by the smaller groups at the two tails, we combined groups 1 and 2 and groups 8 and 9. ANCOVA analysis showed no overall significant differences between the patient groups categorized by GPS scores and baseline peakVO_2 _(Figure 1) or simple and relative difference in peakVO_2 _after training. However, based on linear regression (b = 13.5, p = 0.06) analysis, we observed a trend that higher numbers of increasing alleles (higher GPS) result in a higher baseline peakVO_2_.

**Figure 2:**
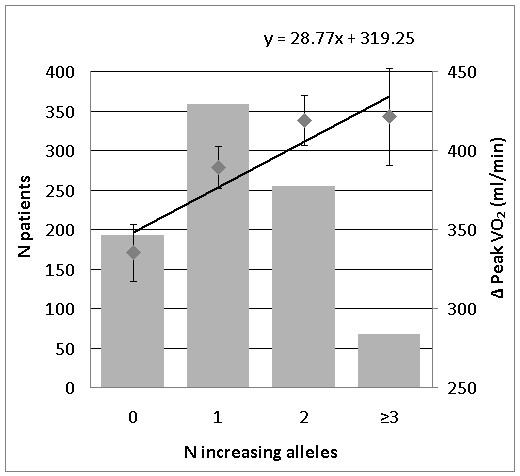
**Genetic predisposition score for 3 polymorphisms associated with the change (ml/min) in peakVO_2 _in the CAREGENE study**. Left Y-axis: Number of patients in each increasing alleles group (bar graph). Right Y-axis: Change in peakVO_2 _(ml/min) for each increasing alleles group (square dots ± SE) corrected for age, gender, height, body mass, and baseline peakVO_2. _X-axis: GPS - Number of increasing alleles. Regression line for the change in peakVO_2 _(ml/min)

**Figure 3:**
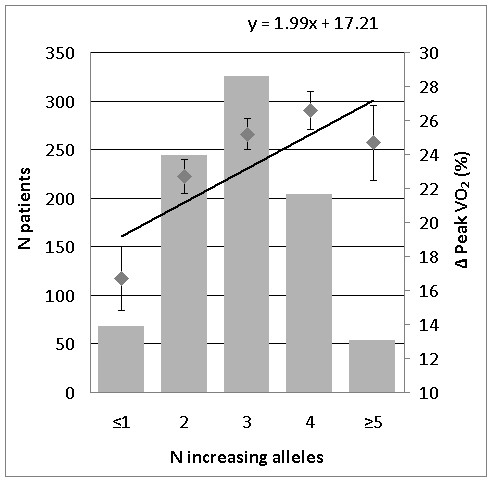
**Genetic predisposition score for 4 polymorphisms associated with relative change (%) in peakVO_2 _in the CAREGENE study**. Left Y-axis: Number of patients in each increasing alleles group (bar graph). Right Y-axis: Relative change in peakVO_2 _(%) for each increasing alleles group (square dots ± SE) corrected for age, gender, height, body mass, and baseline peakVO_2. _X-axis: GPS - Number of increasing alleles. Regression line for the relative change in peakVO_2_

Backward regression analyses revealed three subsets of genes to be associated with baseline peakVO_2 _(ACTN3, Q523R and GR, R23K), simple difference in peakVO_2 _(ACVR2B, rs2268757; CNTF, G-6A and GR, R23K) and relative difference in peakVO_2 _(AMPD1, C34T; ACVR2B, rs2268757; CNTF, G-6A and GR, R23K). Significant differences were found in the models for simple (p < 0.01) and relative difference (p < 0.001) in peakVO_2 _(Figure 2 and 3).

### High vs. Low aerobic capacity groups

**Figure 4:**
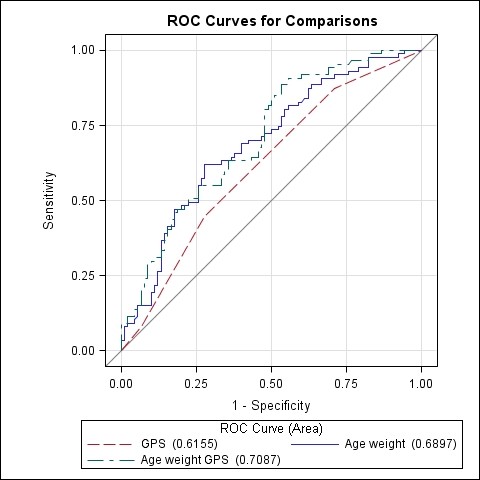
**Overlay of three different models to predict high vs. low responder in peakVO_2 _change (ml/min) after training**. Model GPS (AUC: 0.62; 95% CI: 0.54 - 0.69). Model age and weight (AUC: 0.69; 95% CI: 0.61 - 0.77) Model GPS, age and weight (AUC: 0.71; 95% CI: 0.63 - 0.78)

**Figure 5:**
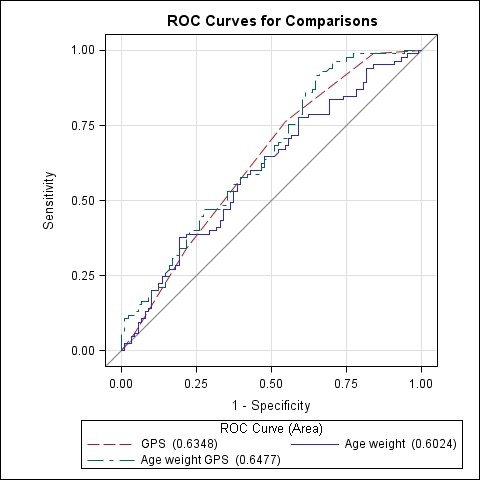
**Overlay of three different models to predict high vs. low responder in relative peakVO_2 _change (%) after training**. Model GPS (AUC: 0.63; 95% CI: 0.56 - 0.71). Model age and weight (AUC: 0.60; 95% CI: 0.52 - 0.69) Model GPS, age and weight (AUC: 0.65; 95% CI: 0.57 - 0.73)

Additional analyses of the high vs. low responder groups were performed for the GPS of the simple and relative difference after training. In the model for simple difference in peakVO_2_, GPS alone was statistically significant to predict a high increase in peakVO_2 _(AUC: 0.62; 95%CI: 0.54-0.69, p < 0.01), (Odds ratio: 1.62; 95%CI: 1.13-2.33) (Figure 4). In the model for relative increase in peakVO_2_, GPS alone was also statistically significant to predict a high relative increase in peakVO_2 _(AUC: 0.63; 95%CI: 0.56-0.71, p < 0.01), (Odds ratio: 1.68; 95%CI: 1.22-2.31) (Figure 5). Both models were comparable to a model with age and weight to predict the simple and relative difference in peakVO_2 _(AUC of 0.69 and 0.60 respectively). When GPS was added to the model with age and weight, the predictive value increased with 2% (simple difference) and 5% (relative difference). However this increase in predictive value was not significantly different from the model with GPS alone or the model with age and weight. Height and gender had no significant contribution to the model and were omitted. A patient with a high GPS has a higher probability to end up in the group with the 10% highest increase in peakVO_2 _values after training and vice versa. The GPS had however no predictive value for the baseline peakVO_2 _for both the literature based model as the own-data model.

## Discussion

Since dynamic resistance training can induce changes in muscle size, fiber type and muscle metabolism [13-16] and can lead to a greater oxygen uptake, we hypothesized that genes coding for these muscular subsystems might also be involved in aerobic power or its change after training through the different pathways of muscular endophenotypes (i.e. structure, growth factors, hormones, ...). We found suggestive associations in polymorphisms of the *GR *gene, *AMPD1 *gene and *CNTF *gene with aerobic capacity or its change after training in CAD patients.

### Single polymorphism associations

#### GR

The mutation in codon 23 results in a change of arginine to lysine. The mutation in R23K polymorphism, which is a part of the ER22/23EK polymorphism complex (as often described in the literature), has been associated with a decreased glucocorticoid sensitivity which can lead to decreased catabolism and less muscle atrophy [33-35]. Van Rossum et al. [36] showed that male ER22/23EK-carriers had a higher muscle mass and strength. In concordance with these results we found that carriers of the minor allele of the R23K polymorphism had a significantly higher increase in aerobic power after three months of training. Although no significant differences in peakVO_2 _were apparent at baseline, a tendency could be observed that carriers of the minor allele had a higher baseline peakVO_2 _(p = 0.08).

#### AMPD1

The C34T mutation results in a premature stop codon of protein synthesis which is the main cause of AMPD deficiency. Fishbein et al. [37] proposed that a deficiency of AMPD causes muscular weakness or cramping after exercise. Rico-Sanz et al. [38] showed that individuals homozygous for the T allele in the C34T polymorphism of the *AMPD1 *gene have diminished exercise capacity and cardiorespiratory responses to exercise in the sedentary state and that the training response of ventilatory phenotypes during maximal exercise is more limited in TT. Due to the small number of individuals in the TT homozygous group, we performed a combined CT + TT vs. CC analysis. We showed that T allele carriers had a significantly lower relative increase in peakVO_2 _after three months of training compared to individuals homozygous for the C allele (p < 0.05) which agrees with earlier findings that the C34T mutation can lead to a decreased exercise capacity and a lower response to exercise (Rico-Sanz 2003).

#### CNTF

Circulating CNTF has trophic effects on neuronal and muscular tissues. Earlier De Mars et al. [39] and Roth et al. [40] hypothesized that, with regard to the *CNTF *G-6A polymorphism, carriers of the minor allele would have lower muscle strength than wild-type carriers due to lower levels of circulating CNTF. However, both research groups were unable to confirm their hypothesis. Subsequently, Roth et al. [40] showed that individuals heterozygous for the G-6A polymorphism possess significantly greater muscle strength and muscle quality at relatively fast contraction speeds compared to homozygous wild-type individuals. Therefore it is plausible that improved muscle strength and quality in these individuals may also result in a more pronounced increase in aerobic power. We showed that A allele carriers of the G-6A polymorphism had a larger increase in aerobic capacity after three months of training (p < 0.01). The homozygous minor allele group had almost 11% additional increase in peakVO_2 _after three months of training compared with the homozygous wild-type group. To our knowledge, aerobic capacity has never been studied in the G-6A polymorphisms of the *CNTF *gene, so it might be that individuals homozygous for the A allele who exhibit higher muscle strength and quality at fast contraction speeds [40] also have a higher aerobic capacity increase, measured on a bicycle treadmill. Unfortunately we do not have muscle strength measurements in this patient group so this hypothesis remains to be confirmed in additional studies.

### Genetic predisposition score

To partially overcome the multiple testing problem and to test for the additive effect of cumulative (response in) VO_2 _uptake- alleles, an 'increasing allele analysis' is performed, as was proposed by studies of Ruiz et al. [29], Santiago et al. [30] and Williams et al. [31]. In calculating a genetic predisposition score (GPS) every polymorphism is considered to have an equal influence on the phenotype, although weighting approaches have also been taken [32]. Based on this hypothesis, every additional occurrence of a 'beneficial' allele for muscular strength would result in an increased peakVO_2_. We constructed an 'increasing allele analysis' for baseline aerobic capacity with 7 polymorphisms, based on the literature. Although no overall significant differences between different allele groups were found, based on linear regression (b = 13.5, p = 0.06) analysis, we observed a trend that a higher GPS results in a higher baseline peakVO_2_. An increase of peakVO_2 _by 13.5 ml/min per allele equals approximately 1% increase in peakVO_2 _with every additional increasing allele.

Based on backward regression analysis of the complete group of polymorphisms, we identified three significant subsets of polymorphisms associated with the three phenotypes; baseline peakVO_2_, simple and relative difference in peakVO_2_. The model for relative change showed remarkable resemblance with the single polymorphism association analysis (only rs2268757 of the *ACVR2B *gene was not significantly associated in the single polymorphism associations). The GPS models for simple and relative difference in peakVO_2 _showed significant associations with the change in peakVO_2 _after training. The GPS for the simple difference showed that patients with a GPS of 0 had an increase of 335.3 ± 18.0 ml/min whereas patients with a GPS of 2 or more would increase with approximately 420 ml/min. The GPS for the relative change showed similar results. GPS of 1 or less had an increase of 16.7% whereas patients with GPS of 4 or more increased their peakVO_2 _with approximately 25%. The findings in both models were confirmed with an analysis of the 10% highest responders versus the 10% lowest responders for both simple difference (ml/min) and relative difference (%) in peakVO_2 _after training. The odds ratio estimate for both models showed that with every additional increasing allele, the chance of belonging to the high responder peakVO_2 _group is 62% and 68% higher for simple and relative response respectively.

In conclusion, although we found some suggestive associations between SNPs with aerobic capacity or its improvement after three months of training, one should carefully interpret the results. The CAREGENE study population is not a random sample of the total population, as they are all Caucasian CAD patients who had been hospitalized before baseline testing (± 8 weeks post-operation). These CAD patients who probably were deconditioned by their disease have proceeded through a spontaneous recovery which might also differ individually. Generalization is therefore limited. It might be that our individuals are also genetically predisposed for CAD and that this patient group might have distorted allele frequencies for the genes under study. However allele frequencies did not differ to the reported CEPH frequencies in the HapMap database. One could speculate that if the identified gene variants are not specifically related to the CAD-status of the patients, and if underlying pathways that drive the adaptations do not differ between CAD and healthy individuals, that these might also play a role in exercise-induced changes in aerobic capacity in healthy individuals.

Literature search at the beginning of the study led to a selection of genes and polymorphisms in which associations were found with muscle structure or muscle function. We are aware that the field is evolving quickly and 'new' candidate gene variants have become available in the mean time. We hypothesized that if patients had better muscular performance by mutations of their genes they might also have a better aerobic capacity or the ability to improve more beneficially. This could be generated by a better local oxygen extraction by the muscles, which leads to a higher peakVO_2_. In a substudy on 80 patients we found a correlation of r = 0.60 between baseline quadriceps isometric and isokinetic muscle force and baseline peakVO_2 _and a correlation of r = 0.32 between change in peakVO_2 _and change in isometric muscle force [20]. Although these phenotypic correlations have not been decomposed into genetic versus environmental covariation between the endurance and strength phenotypes, they are however indicative for at least partial pleiotrophic gene action. For more closely related phenotypes e.g. walking speed and lower leg power, substantial genetic correlations have been reported [41]. Although we describe some pathways within our associations, the lack of an objective measurement of muscle force or function makes it hard to confirm these hypotheses. In addition one could also question if these genes for muscular structure and function are 'good endophenotypes' for improved peakVO_2 _as some studies have indicated that the 'increasing allele' for endurance is not necessarily the same as for maximal strength. With regard to genetic influence on aerobic capacity and/or its improvement after training, more research is definitively needed with additional muscular testing.

Furthermore we set the significance level at p < 0.05, however when applying Bonferroni correction for multiple testing none of our results would reach significance. This study therefore also included a genotypic predisposition score analysis, in which additive effects on seven polymorphisms were studied, as well as a data-driven GPS analysis was performed. Although meaningful phenotypic correlations between muscle strength and endurance performance in this specific population do suggest underlying pleiotrophic gene actions, the contribution of each specific genetic variant is expected to be small.

## Conclusion

Suggestive associations were found for changes in aerobic capacity and single polymorphisms of the *CNTF, AMPD1 *and *GR *gene and a genetic predisposition score showed a significant predictive value for this GPS to estimate an individuals' potential to belong to the higher peakVO_2 _responder group, or inversely to predict the risk of belonging to the low responder peakVO_2 _group. Although no strong associations were found, it is reasonable to assume that mutations in genes coding for muscular structure and function can lead to changes in aerobic capacity.

## Competing interests

The authors declare that they have no competing interests.

## Authors' contributions

TT analyzed and interpreted the data, performed statistical analysis, and drafted the manuscript. MT performed statistical analysis, assisted with interpretation of the data. SO and RB assisted with collection and analyses of the data. GM acquired the data and performed genetic analyses. DS acquired and interpreted the exercise data. VC assisted with interpretation of the data. RF and LV: conceived and designed the research. All authors read, approved and contributed to the manuscript.

## Supplementary Material

Additional file 1 **The selected increasing alleles for calculation of the GPS score of the increasing allele analysis**. For the 'increasing allele' analysis a selection of 7 polymorphisms was made based on recent literature. The increasing allele is indicated here for each selected polymorphism. Click here for file

Additional file 2 **Genotype-phenotype association analysis for muscular subsystem gene polymorphisms and baseline aerobic power and changes after training in the CAREGENE study (without correction for baseline peakVO_2_)**. As we might be overcorrecting the increase in peakVO_2 _by using baseline peak VO_2 _values as a covariate, the results of Table 3 are shown here without correction for baseline peakVO_2_. Click here for file
