# Supplementary material for: A genetic predisposition score for muscular endophenotypes predicts the increase in aerobic power after training: the CAREGENE study
Source: BMC Genet. 2011 Oct 3;12:84. doi: 10.1186/1471-2156-12-84 (PMC3193032; doi:10.1186/1471-2156-12-84)
Supplement: Additional file 1 — The selected increasing alleles for calculation of the GPS score of the increasing allele analysis. For the 'increasing allele' analysis a selection of 7 polymorphisms was made based on recent literature. The increasing allele is indicated here for each selected polymorphism. [file 1471-2156-12-84-S1.DOC]

**Additional file 1. The selected increasing alleles for calculation of the GPS score of the increasing allele analysis**

The ‘increasing allele’ is underlined for each polymorphism.

Alpha-actinin 3 (*ACTN3)* R577X

Myosine light chain kinase (*MLCK)* C49T

Insulin-like growth factor 2 (*IGF-II)* ApaI, G-allele

Glucocorticoid receptor (*GR)* R23K

Interleukin 15 receptor alpha (*IL15Ra)* BstnI, G-allele

Ciliary Neurotrophic Factor Receptor *(CNTFR)* C-1703T and C174T
