# Supplementary material for: A genetic predisposition score for muscular endophenotypes predicts the increase in aerobic power after training: the CAREGENE study
Source: BMC Genet. 2011 Oct 3;12:84. doi: 10.1186/1471-2156-12-84 (PMC3193032; doi:10.1186/1471-2156-12-84)
Supplement: Additional file 2 — Genotype-phenotype association analysis for muscular subsystem gene polymorphisms and baseline aerobic power and changes after training in the CAREGENE study (without correction for baseline peakVO2). As we might be overcorrecting the increase in peakVO2 by using baseline peak VO2 values as a covariate, the results of Table 3 are shown here without correction for baseline peakVO2. [file 1471-2156-12-84-S2.DOC]

**Table S1. Genotype-phenotype association analysis for muscular subsystem gene polymorphisms and baseline aerobic power and changes**

**after training in the CAREGENE study (without correction for baseline peakVO_2_)**

| **Gene** | **Polymorphism** | **Allele** | **Frequency N (%)** | **VO_2_pre (ml/min)** | **p-value** | **Δ VO_2_ (ml/min)** | **p-value** | **VO_2_ (%)** | **p-value** |
| --- | --- | --- | --- | --- | --- | --- | --- | --- | --- |
| **AMPD1** | C34T / rs17602729 | CC | 652 (71) | 1711 ± 15 | p=0.40 | 397 ± 10 | p=0.09 | 24.9 ± 0.7 | p=0.03 |
|  |  | CT + TT | 263 (29) | 1734 ± 23 |  | 365 ± 16 |  | 22.1 ± 1.1 |  |
|  |  |  |  |  |  |  |  |  |  |
| **CNTF** | G-6A / rs1800169 | GG | 664 (73) | 1724 ± 15 | p=0.43 | 368 ± 10 | p=0.0009 | 22.9 ± 0.7 | p=0.002 |
|  |  | GA | 226 (25) | 1701 ± 25 |  | 418± 17 |  | 25.5 ± 1.2 |  |
|  |  | AA | 21 (2) | 1801 ± 81 |  | 526± 53 |  | 34.8± 3.7 |  |
|  |  |  |  |  |  |  |  |  |  |
| **GR** | R23K / rs6190 | GG | 857 (94) | 1715 ± 13 | p=0.08 | 383 ± 9 | p=0.04 | 23.9 ± 0.6 | p=0.15 |

AMPD1, Adenosine monophosphate deaminase; CNTF , Ciliary neurotrophic factor; GR, Glucocorticoid receptor

Mean ± SE, corrected for gender, age, height and weight
